# Confirming the Cognition of Rising Scores: Fox and Mitchum (2013) Predicts Violations of Measurement Invariance in Series Completion between Age-Matched Cohorts

**DOI:** 10.1371/journal.pone.0095780

**Published:** 2014-05-07

**Authors:** Mark C. Fox, Ainsley L. Mitchum

**Affiliations:** 1 Department of Psychology, University of Richmond, Richmond, Virginia, United States of America; 2 Department of Psychology, Florida State University, Tallahassee, Florida, United States of America; Nathan Kline Institute and New York University School of Medicine, United States of America

## Abstract

The trend of rising scores on intelligence tests raises important questions about the comparability of variation within and between time periods. Descriptions of the processes that mediate selection of item responses provide meaningful psychological criteria upon which to base such comparisons. In a recent paper, Fox and Mitchum presented and tested a cognitive theory of rising scores on analogical and inductive reasoning tests that is specific enough to make novel predictions about cohort differences in patterns of item responses for tests such as the Raven’s Matrices. In this paper we extend the same proposal in two important ways by (1) testing it against a dataset that enables the effects of cohort to be isolated from those of age, and (2) applying it to two other inductive reasoning tests that exhibit large Flynn effects: Letter Series and Word Series. Following specification and testing of a confirmatory item response model, predicted violations of measurement invariance are observed between two age-matched cohorts that are separated by only 20 years, as members of the later cohort are found to map objects at higher levels of abstraction than members of the earlier cohort who possess the same overall level of ability. Results have implications for the Flynn effect and cognitive aging while underscoring the value of establishing psychological criteria for equating members of distinct groups who achieve the same scores.

## Introduction

The trend of rising scores on intelligence tests across cohorts, known as the Flynn Effect [1], [2], has generated considerable controversy as to the comparability of variation within and between time periods [3]. Statistical methods such as confirmatory factor analysis have already revealed that a given latent variable score predicts different scores on the same tests in different cohorts [4], [5]. However, as informative as such studies are, purely psychometric methods do not provide the much-needed psychological basis for comparing between cohorts, namely, criteria for sameness that are logically distinct from variation itself. By relying on psychometric criteria alone to establish comparability, abilities are allowed to be defined by variation itself, which invites a host of conceptual problems, one of the more telling of which is the veritable disappearance of abilities that do not vary within one time period [6], [7].

In contrast to purely psychometric criteria, descriptions of the cognitive processes that mediate selection of item responses do provide such a basis because they remain meaningful even in the absence of variation, as illustrated by process-oriented models of task performance that are comprehensible in the context of one person (e.g., production models). An analysis of test items aimed at identifying which cognitive processes must be completed to select the correct responses by any individual in *any* conceivable population leads to predictions about which item features may be expected to elicit variations in item-response probabilities in different populations based on hypotheses about the distribution of processes within these populations. Models of latent variables defined by these item features can be tested against datasets containing item responses from multiple populations and followed up with additional analyses to determine whether the variables vary similarly in both [7].

In this paper we adopt this process-oriented psychometric framework to test a cognitive theory of cohort differences in analogical and inductive reasoning [7] against the Letter Series and Word Series data in the readily available Long Beach Longitudinal Dataset [8], [9]. Fox and Mitchum’s [7] proposal has already received empirical support from a dataset in which cohort was confounded with age group. However, given that performance on inductive reasoning tests declines with age [10], age-matched comparisons allow for more compelling tests of predictions about cohort differences. The present dataset enables the same basic predictions to be tested by comparing at least two cohorts that are matched for age. Zelinski and Kennison [8] have already established that the dataset reveals a large cohort effect consistent with the more general trend of rising intelligence test scores over time. In the present paper, we test relatively specific predictions about violations of measurement invariance between cohorts that build significantly on Zelinski and Kennison’s [8] work.

### Cohort Differences in Inductive Reasoning

During the 20^th^-century, scores on intelligence rose considerably from one cohort to another at a rate of about three IQ points per decade [11]. What is most striking about the Flynn effect is that it is most pronounced on the very kinds of tests that were once believed to be relatively insusceptible to cultural influence [12], namely, tests that call for abstract analogical and inductive reasoning.

In a recent paper, Fox and Mitchum [7] proposed that members of more recent cohorts utilize more abstract concepts when reasoning inductively than do their predecessors; that is, they are more able to map objects that are similar only in an abstract (as opposed to concrete) sense. Throughout this paper we refer to this ability as *abstraction.* At its heart, Fox and Mitchum’s [7] proposal is a relatively straightforward extension of extant literature on the cognition of matrix reasoning where it has been shown that items are more difficult to solve when the objects that must be mapped to one another are physically dissimilar [13], [14].

What distinguishes Fox and Mitchum’s [7] proposal is their emphasis on the relational status of between-subjects variables: they do *not* reflect intrinsic properties of persons [15], [16], but rather the relation between an instrument and the distribution of psychological processes and competencies within a whole population. The implication is that psychological sources of variation within commonly studied (e.g., contemporary) populations cannot be taken for granted to be the same as sources of variation in other (e.g., earlier) populations even if the same instruments elicit variation in both. For example, individual differences in the level of abstraction at which individuals use concepts and categories may vary substantially more between cohorts than it does within cohorts even if the distribution of scores is similar in both populations.

If it can be assumed that performance on a test is determined by other abilities in addition to the level of abstraction at which persons can map objects, then Fox and Mitchum’s [7] theory makes fairly straightforward predictions about differences between cohorts in the relative contributions of abstraction and these other abilities to any given overall score. Specifically, members of more recent cohorts should evince higher levels of abstraction than members of earlier cohorts who achieve the same overall score. At the level of individual items within a test, the prediction is that members of more recent cohorts should outperform members of earlier cohorts who achieve the same overall score to the extent that selecting the correct response to an item depends on mapping dissimilar objects.

### A Confirmatory Model for Predicting Violations of Measurement Invariance

Our starting point for modeling the predicted violation of measurement invariance between cohorts is the Multidimensional Random Coefficients Multinomial Logit Model (MRCMLM) [17], a highly flexible family of models that enables cognitive theories of the item response process and/or individual differences to be expressed within the formalism of an item response model. The probability of a correct response is
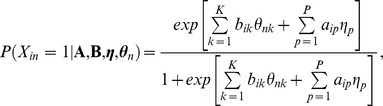
where 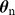 is the ability vector of person *n,* **A** is a design matrix that defines the decomposition of items into basic parameters *η_p_* according to a cognitive theory of item difficulty, and **B** is a score matrix that defines the variation in response accuracy probabilities across persons elicited by variations in item features. The most unique characteristic of this generalization from a philosophy-of-science standpoint is its accommodation of highly confirmatory models that are constrained to allow relatively few open parameters based on a cognitive theory of the item response process.

Rijmen, de Boeck, and Leuven [18] propose a unique model subsumed by the MRCMLM, the Random Weights Linear Logistic Test Model (RWLLTM), that is especially relevant to formalizing predictions within this paper (see 18 for more details). This model allows a predicted violation of measurement invariance between cohorts to be conceptualized as a difference between cohorts in the relative contributions to overall performance of two abilities: (1) ability as typically defined (the intercept, or random effect of persons across items) and (2) the hypothesized between-cohort ability of abstraction, defined as between-person variation elicited by the item features predicted to discriminate between members of different cohorts according to Fox and Mitchum’s [7] proposal.

Under the null hypothesis of measurement invariance, members of earlier cohorts and members of later cohorts who achieve the same score on the intercept should also achieve the same score on abstraction. However, if as we predict, the superior performance of members of more recent cohorts is concentrated in higher levels of abstraction, then members of more recent cohorts should score higher on abstraction than members of earlier cohorts who achieve the same score on the intercept.

### Cognitive Decomposition of Letter Series and Word Series Tests

A cognitive analysis of series completion items is needed to decompose items into features that elicit variation in abstraction. The goal of series completion tests is to determine which object comes next in a series of objects, all of which belong to a common sequence that is assumed to be known by test-takers such as the alphabet. The Letter and Word Series tests [19] both fall into this category. In Letter Series, test-takers are presented with a series of letters of the alphabet, and must choose which letter comes next out five response choices. Word Series is the same except that it features months of the year, and to a lesser extent, days of the week, instead of letters of the alphabet. For the purpose of this analysis, we assume test-takers have all mastered the alphabet to a greater or lesser extent, and are capable of reciting the months of the year and the days of the week in order. We further assume that there are no differences between cohorts in these abilities.

Although it may not be readily apparent, Letter Series and Word Series items are very similar in principle to matrix reasoning items, which can be seen by decomposing them into complete iterations of their constituent patterns. These iterations can be regarded as analogs to corresponding ordered sequences within the alphabet or calendar. For example, the item, *abbcddeff_* becomes the analogy, *ab:abb::cd:cdd::ef:_.* Of course, the principal challenge of solving a series completion item is not knowing where one analog ends and the next begins, as we consider below. Nevertheless, this formalism provides a fairly objective and uniform basis for describing the relevant features of items for modeling predicted variations in the item response process.

There are two basic ways that Letter and Word Series items vary with respect to the overall level of abstraction at which objects must be mapped: presence of a constant, and level at which remaining objects must be mapped between analogs. Many items contain a “constant,” an object that remains unchanged in every analog of a series. For example, the series, *aybycydy_* contains the constant, *y.* Constants are, by definition, concrete because they are their own roles; that is, they can be mapped from one analog to another without the need to infer concepts more abstract than themselves. In most items, constants are combined with more abstract relations. In fact, the presence of constants would not be expected to elicit much variation in series completion were in not that, as noted above, the boundaries between analogs in these items are not specified for the test-taker (as they are in matrix reasoning tests and most other inductive reasoning tests). By helping to demarcate analogs, constants are expected to reduce item difficulty by making it easier to map objects that serve roles in more abstract relations. Thus, the absence of a constant is a feature of more difficult items for the abstraction variable.

The second way in which items vary with respect to overall level of abstraction is the level of abstraction at which remaining objects (those other than constants) are mapped between analogs. There are two levels: level 1 and level 2, with level 2 subsuming level 1. Level 1 roles are letters of the alphabet (*letters*), months of the year (*months*), and days of the week (*days*), which enable mapping of objects according to their positions within the alphabet, year, or week respectively. Level 2 roles are hierarchical orderings of objects called *ordered sequences* that enable mapping of objects according to their serial positions within analogs.

It is more informative to demonstrate than explain the distinction between level 1 and level 2 roles within the tests. In Letter Series, test-takers must make use of the role, *letter,* which can occupy any one of 26 positions, as a place-keeper between analogs. For example, inferring the next letter in the series, *mqnqoqpqqq_,* requires knowing the serial positions within the concept, *letter,* but does not require inferring any higher level relations with more abstract serial positions. The roles, *month,* which can occupy any of 12 positions, and *week,* which can occupy any of seven, serve the same purpose within Word Series.

At level 2, test-takers must make use of ordered sequences of objects (usually one, two, or three objects) within which different letters perform the same role in different analogs defined by serial position. Consider the series, *abbacddceff_,* which is transformed into *ab:abba::cd:cddc::ef:eff_.* Within each ordered sequence of three letters, the second serial position is repeated immediately, and the first serial position is repeated at the end of the ordered sequence such that the serial positions are *1, 2, 2, 1* in every analog. Items that require the use of ordered sequences are the most abstract because there is no concrete similarity between objects that serve the same role in different analogs. Notice that *ordered sequence* subsumes *letter* because it is not possible in principle to use the former without the latter.

The classifications for all 30 Letter Series and 30 Word Series items according to this decomposition are presented in Table 1. Item in both tests are isomorphic by item number, leading to identical classifications between tests. The translation of these item classifications into the confirmatory item response model is described below.

**Table 1:** Decomposition and Classification of Items.

Test	Item number	Roles	Presence of constant	Level of abstraction
Letter Series	1	Letter(*a…z*), ordered sequence of two letters(serial position 1(*a…z*), 2(*a…z*))	No	2
	2	*x, y,* letter(*a…z*)	Yes	1
	3	Letter(*a…z*), ordered sequence of three letters(serial position 1(*a…z*), 2(*a…z*), 3(*a…z*))	No	2
	4	*x y z,* letter(*a…z*), ordered sequence of three letters(serial position 1(*a…z*), 2(*a…z*), 3(*a…z*))	Yes	2
	5	*a b,* letter(*a…z*)	Yes	1
	6	*x z y,* letter(*a…z*)	Yes	1
	7	*c,* letter(*a…z*), ordered sequence of two letters(serial position 1(*a…z*), 2(*a…z*))	Yes	2
	8	*c b a*	Yes	1
	9	*m,* letter(*a…z*), ordered sequence of x letters(serial position 1(*a…z*), 2(*a…z*), 3(*a…z*)….)	Yes	2
	10	Letter(*a…z*), ordered sequence of two letters(serial position 1(*a…z*), 2(*a…z*))	No	2
	11	*c d,* letter(*a…z*), ordered sequence of two letters(serial position 1 or 2)	Yes	2
	12	Letter(*a…z*)	No	1
	13	*a b c,* letter(*a…z*), ordered sequence of letters(serial position 1(*a…z*), 2(*a…z*), 3(*a…z*)….)	Yes	2
	14	Letter(*a…z*), ordered sequence of three letters(serial position 1(*a…z*), 2(*a…z*), 3(*a…z*))	No	2
	15	Letter(*a…z*), ordered sequence of three letters(serial position 1(*a…z*), 2(*a…z*), 3(*a…z*))	No	2
	16	*n o,* letter(*a…z*), ordered sequence of three letters(serial position 1(*a…z*), 2(*a…z*), 3(*a…z*))	Yes	2
	17	Letter(*a…z*), ordered sequence of two letters(serial position 1(*a…z*), 2(*a…z*))	No	2
	18	Letter(*a…z*)	No	1
	19	Letter(*a…z*), ordered sequence of two letters(serial position 1(*a…z*), 2(*a…z*))	No	2
	20	*a x b y c z*	Yes	1
	21	Letter(*a…z*), ordered sequence of two letters(serial position 1(*a…z*), 2(*a…z*))	No	2
	22	Letter(*a…z*), ordered sequence of three letters(serial position 1(*a…z*), 2(*a…z*), 3(*a…z*))	No	2
	23	Letter(*a…z*), ordered sequence of three letters(serial position 1(*a…z*), 2 (*a…z*), 3(*a…z*))	No	2
	24	Letter(*a…z*)	No	1
	25	Letter(*a…z*), ordered sequence of three letters(serial position 1(*a…z*), 2(*a…z*), 3(*a…z*))	No	2
	26	Letter(*a…z*), ordered sequence of two letters(serial position 1(*a…z*), 2(*a…z*))	No	2
	27	Letter(*a…z*), ordered sequence of three letters(serial position 1(*a…z*), 2(*a…z*), 3(*a…z*),4(*a…z*), 5(*a…z*))	No	2
	28	Letter(*a…z*)	No	1
	29	Letter(*a…z*), ordered sequence of three letters(serial position 1(*a…z*), 2(*a…z*), 3(*a…z*))	No	2
	30	Letter(*a…z*), ordered sequence of three letters(serial position 1(*a…z*), 2(*a…z*), 3(*a…z*))	No	2
Word Series	1	Month(*Jan…Dec*), ordered sequence of two months(serial position1(*Jan…Dec*), 2(*Jan…Dec*))	No	2
	2	*Sun, Mon,* month(*Jan…Dec*)	Yes	1
	3	Month(*Jan…Dec*), ordered sequence of three months(serial position 1(*Jan…Dec*),2(*Jan…Dec*), 3(*Jan…Dec*))	No	2
	4	*Sun Mon Tue,* month(*Jan…Dec*), ordered sequence of three months (serial position1(*Jan…Dec*), 2(*Jan…Dec*), 3(*Jan…Dec*))	Yes	2
	5	*Jan Feb,* month(*Jan…Dec*)	Yes	1
	6	*Thu Fri Sat,* month(*Jan…Dec*)	Yes	1
	7	*Tue,* month(*Jan…Dec*), ordered sequence of two months(serial position 1(*Jan…Dec*), 2(*Jan…Dec*))	Yes	2
	8	*Mar Feb Jan*	Yes	1
	9	*Wed,* month(*Jan…Dec*), ordered sequence of varying number ofmonths(serial position 1(*Jan…Dec*), 2(*Jan…Dec*), 3(*Jan…Dec*)….)	Yes	2
	10	Month(*Jan…Dec*), ordered sequence of two months(serial position 1(*Jan…Dec*), 2(*Jan…Dec*))	No	2
	11	Month(*Jan…Dec*), ordered sequence of two months(serial position 1(*Jan…Dec*), 2(*Jan…Dec*))	No	2
	12	Month(*Jan…Dec*)	No	1
	13	*Jan Feb Mar,* month(*Jan…Dec*), ordered sequence of varying number of months(serial position 1(*Jan…Dec*), 2(*Jan…Dec*), 3(*Jan…Dec*)….)	Yes	2
	14	Month(*Jan…Dec*), ordered sequence of three months(serial position1(*Jan…Dec*), 2(*Jan…Dec*), 3(*Jan…Dec*))	No	2
	15	Month(*Jan…Dec*), ordered sequence of two months(serial position1(*Jan…Dec*), 2(*Jan…Dec*))	No	2
	16	*Thu Fri,* month(*Jan…Dec*), ordered sequence of three months(serial position 1(*Jan…Dec*), 2(*Jan…Dec*), 3(*Jan…Dec*))	Yes	2
	17	Month(*Jan…Dec*), ordered sequence of two months(serial position 1(*Jan…Dec*),2(*Jan…Dec*))	No	2
	18	Month(*Jan…Dec*)	No	1
	19	Month(*Jan…Dec*), day(*Sun…Sat*), ordered sequence of two months (serial position1(*Jan…Dec*), 2(*Jan…Dec*)), ordered sequence of two days(serial position 1(*Sun…Sat*),2(*Sun…Sat*))	No	2
	20	*Jan Thu Feb Fri Mar Sat*	Yes	1
	21	Month(*Jan…Dec*), ordered sequence of two months(serial position1(*Jan…Dec*), 2(*Jan…Dec*))	No	2
	22	Month(*Jan…Dec*), day(*Sun…Sat*), ordered sequence of three months (serial position1(*Jan…Dec*), 2(*Jan…Dec*), 3(*Jan…Dec*)), ordered sequence of three days (serial position1(*Sun…Sat*), 2(*Sun…Sat*), 3(*Sun…Sat*))	No	2
	23	Month(*Jan…Dec*), ordered sequence of three months(serial position 1(*Jan…Dec*),2(*Jan…Dec*), 3(*Jan…Dec*))	No	2
	24	Month(*Jan…Dec*), day(*Sun…Sat*)	No	1
	25	Month(*Jan…Dec*), ordered sequence of three months(serial position1(*Jan…Dec*), 2(*Jan…Dec*), 3(*Jan…Dec*))	No	2
	26	Month(*Jan…Dec*), ordered sequence of two months(serial position1(*Jan…Dec*), 2(*Jan…Dec*))	No	2
	27	Month(*Jan…Dec*), ordered sequence of five months(serial position1(*Jan…Dec*), 2(*Jan…Dec*), 3(*Jan…Dec*), 4(*Jan…Dec*), 5(*Jan…Dec*))	No	2
	28	Month(*Jan…Dec*)	No	1
	29	Month(*Jan…Dec*), ordered sequence of three months (serial position1(*Jan…Dec*), 2(*Jan…Dec*), 3(*Jan…Dec*))	No	2
	30	Month(*Jan…Dec*), ordered sequence of three months(serial position1(*Jan…Dec*), 2(*Jan…Dec*), 3(*Jan…Dec*))	No	2

Before continuing, it is worth emphasizing that given the background assumptions such as the prior knowledge of test-takers, the 60 items do not lend themselves to classifications that are substantially different than the one we have presented. Although arguments can be made for making slight alterations to our system, for example, expanding or collapsing the categories, any reasonable a priori classification of the same items in terms of level of abstraction would be highly correlated with, and closely resemble, the one presented in Table 1.

It is also noteworthy that Letter and Word Series items, like the items of most tests whose designs were not guided by a cognitive theory, do not vary in perfect harmony with level of abstractness as envisaged by Fox and Mitchum’s [7] proposal. This leads to several potential limitations, for example, that the test may artificially constrain the range of scores on abstraction. In fact, the most abstract relations within the test are considerably less abstract than the most abstract relations in other tests such as matrix reasoning. The use of letters and months renders the items relatively concrete by definition for anyone familiar with these concepts. See Meo, Roberts, and Marucci [20] and Fox and Mitchum’s [7] discussion on p. 984 of the same paper.

The test may also confound abstraction and other item features that are incidental to hypotheses. For example, up to this point, literature on the cognition of series completion has mirrored the matrix reasoning literature in focusing almost exclusively on differences in working memory load as the source of variations in item difficulty, and differences in working memory capacity as sources of individual differences in ability [21]–[23]. Working memory load of items in these studies is defined in a way that confounds this variable with the level of abstraction at which concepts and categories must be inferred as we define it above. Upon close inspection, such definitions of working memory load are bound to the populations in which they were operationalized because they rest on the implicit assumption that anyone who possesses sufficient working memory capacity necessarily possesses the ability to identify abstract concepts and categories. Even if this assumption were reasonable within the relatively homogeneous populations in which these studies were conducted, such a definition divests working memory of its ordinary meaning when applied to broader populations including individuals who are expected to use concepts poorly irrespective of working memory capacity. In short, a working memory interpretation of our classifications in the context of cohort differences amounts to making precisely the kind of generalization from within-cohort study to between-cohort theorizing that we are endeavoring to avoid.

## Methods

### Dataset

Participants and item responses were taken from the readily available Long Beach Longitudinal dataset from three panels begun in 1978, 1994, and 2000. At any given time of testing, the dataset is comprised primarily of adults older than 60 years (and as old as 100 years), but also includes a smaller sample of younger adults (as young as 28 years). The sample is roughly half female and fairly well educated, irrespective of age or cohort, with an average of 12 to 14 years of education depending on how groups are defined. More specific details about the design of the Long Beach Longitudinal Study and collection of data can be found in Zelinski and Kenisson [8], Zelinski and Burnight [24], and Zelinski and Lewis [9].

Because our aim is to compare cohorts that are matched on age rather than to model individual change over time, we use item responses only from participants’ first test session, which enables us to maximize the number of data available for analysis while eliminating concerns about the effects of practice across testing sessions. The total number of individuals for whom initial data are available is *n* = 2,169.

In order to isolate the effects of cohort from age group, it is necessary to have at least two cohorts that are matched on age at the time of testing. Note that in many cases it was necessary to calculate birth year from age at time of testing, which can be expected to have resulted in errors of no more than one year assuming that age was reported accurately. For the sake of simplicity, we wanted to avoid defining two cohorts with overlapping birth years as did Zelinski and Kennison [8]. A cross-tabulation revealed that a fairly large span of overlapping ages could be obtained in a straightforward way by defining one cohort that terminates in 1920 (cohort 1), and a second that commences in 1921 and terminates in 1940 (cohort 2). The ages at the time of testing common to both cohorts range from 58 to 79 years, meaning that it was necessary to omit participants from cohort 1 who were older than 79 years at the time of testing, and participants from cohort 2 who were younger than 58 years at the time of testing to assure complete overlap of ages. As a result of these omissions, cohort 1 necessarily commences in 1899, roughly 10 years after the birth of the oldest participant in the study. Cohorts 1 (*n* = 511) and 2 (*n* = 723) are roughly matched on age at the time of testing (cohort 1: *M* = 69.65, *SD = *6.24; Cohort 2: *M* = 68.78, *SD* = 5.49).

Responses of individuals who are omitted to create age-matched cohorts can still, in principle, help to disconfirm predictions about cohort differences, irrespective of the confound these individuals introduce between cohort and age. For this reason, individuals who were too old to be included in cohort 1 (*n* = 483) or too young to be included in cohort 2 (*n* = 79) for the age-matched comparison are included in a follow-up analysis that is not intended to distinguish the effects of age and cohort. We also consider a third group, cohort 3, comprised of 373 individuals born from 1941 through 1971, who were considerably younger at the time of testing than members of cohorts 1 or 2 (*M* = 44.97, *SD* = 9.06).

### Model Specification

The model envisages overall ability on the Series tests as the joint contribution of the intercept (ability as normally defined) and abstraction as defined by the decomposition of item features described above. The classifications in Table 1 were used to construct the design and score matrices shown in Table 2.

**Table 2:** Design and Score Matrices.

	Design matrix				Score matrix	
Item type	Intercept	P1	P2	P3	Intercept	Abstraction
Constant, level 1	1	0	0	0	1	0
Constant, level 2	1	0	1	0	1	1
No constant, level 1	1	1	0	0	1	1
No constant, level 2	1	1	1	1	1	2

*Note.* P = parameter.

The two intercepts in Table 2, one in the design matrix and one in the score matrix, correspond to the effects of individual items and persons respectively, irrespective of predictions about cohort differences. By themselves, the intercepts can be conceptualized as an ordinary Rasch model of performance representing the unidimensional variation that is common across cohorts.

The remaining columns are the formalization of predictions about differences between cohorts. The columns of the design matrix represent the basic parameters for decomposition of items into four types with respect to level of abstraction. Parameter 1 represents presence versus absence of a constant (irrespective of level of relation), parameter 2 represents a level 1 versus level 2 relation (irrespective of whether there is a constant), and parameter 3 represents the combination of both presence versus absence of a constant and level 1 versus level 2 relation. The score matrix represents the between-persons variation elicited by the decomposition of item features in the design matrix (i.e., between-persons variation predicted to be observed primarily between cohorts). Notice that the score matrix does not distinguish between the second and third type of item in the design matrix, which simply means that these items are treated as eliciting the same variation between persons.

In an effort to simplify the presentation of analyses and results, we model the data for Letter and Word Series tests together, although the same basic pattern of results is observed when modeling either test in isolation. A random effects component for test is added to the model to allow for variation between tests that is not predicted by the theory.

Our approach to testing for predicted violations of measurement invariance is to conduct dedicated tests after estimating the model rather than building such tests into the model itself. In fact, the absence of any information about cohort membership within the model itself renders scatterplots of ability estimates especially compelling when they nevertheless reveal stratification of groups by individuals [7].

## Results

### Flynn Effect

Before attempting to account for a Flynn effect, it is first necessary to establish that there is indeed a Flynn effect. Zelinski and Kennison [8] have already established the existence of a Flynn effect within the present dataset for Letter and Word Series tests. Nevertheless, because we define cohorts somewhat differently, we present an estimate of the Flynn effect according to our own classification. Like Zelinski and Kennison [8], we define the effect in terms of ordinary Rasch scores. The reason for using Rasch scores rather than raw scores is that there are too many missing responses for observed raw scores to be accurate reflections of raw scores had all responses been present. That the data fit an ordinary Rasch model fairly well, and the Rasch model reduces to raw score in principle [25], suggests that the Flynn effect defined by Rasch scores would be virtually indistinguishable from the Flynn effect defined by raw scores had complete raw scores been available.

The difference between cohort 1 and cohort 2 corresponds to an effect size of *d = *.38 or the equivalent of about 6 IQ points. This effect size is in keeping with the effect size reported by Zelinski and Kennison [8] as well as Flynn’s [11] generalization that the Flynn effect corresponds to about 3 IQ points per decade.

### Model Assessment

The confirmatory model was estimated using marginal maximum likelihood. Because results do not (and should not) depend on using only responses of members of the age-matched cohorts to estimate the model, parameters were estimated using responses of every participant (*n* = 2,169), irrespective of age or cohort.

Compatibility of data and model is assessed by examining the weighted and unweighted mean-squares and T-values of the items and basic parameters. When the data are exactly as predictable as a model assumes, expected values are 1 and 0 for mean-squares and T-values respectively. Lower values indicate that the data are more predicable than the model assumes–there is redundancy in the model–and higher values indicate that data are less predictable than the model assumes.

The model fits the data fairly well. With respect to the intercept (the ordinary ability component of the model), almost all items are found to have weighted and unweighted mean-squares between.5 and 1.5. As for the component of the model representing abstraction or predicted between-cohort variation, the values of the basic parameters also reveal good fit, as shown in Table 3. Note that some degree of unexplained variation is to be expected as we are predicting violations of measurement invariance between cohorts, but have not specified the cohort membership of participants within the model.

**Table 3:** Basic Parameter Estimates for Abstractness Component of Model with Fit Statistics.

		Weighted fit		Unweighted fit	
Parameter	Estimate	Mean-square	T	Mean-square	T
P1	–0.67	1.05	1.60	1.04	1.00
P2	–0.67	1.04	1.30	1.03	0.90
P3	00.87	1.02	0.60	1.00	0.00

*Note.* Mean-square values of 1 and T-values of 0 indicate ideal fit.

### Testing the Predicted Violation of Measurement Invariance

In the next stage of analysis we test the prediction that, given the model, members of later cohorts achieve higher scores on the abstraction latent variable than do members of earlier cohorts who achieved the same score on the intercept. A regression model was tested in which abstraction score is the dependent variable predicted by the intercept, cohort membership, and a term representing the interaction between the intercept and cohort membership. An effect of cohort membership implies a violation of measurement invariance because it reveals that information about cohort membership improves predictions of abstraction above and beyond the intercept alone. An effect of the interaction term implies a more complex violation of measurement invariance in which the relationship between cohort and abstraction varies as a function of the intercept.

The analysis revealed a significant effect of cohort, confirming the prediction that members of cohort 2 achieve higher abstraction scores given their intercepts than do members of cohort 1, *t*(1) = 6.37, *p*<.001. The interaction term was marginally significant *t*(1) = 1.94, *p* = .05, suggesting the difference in abstraction between cohorts is greater at higher levels of the intercept, although this effect was not predicted and should be interpreted accordingly.

**Figure 1:**
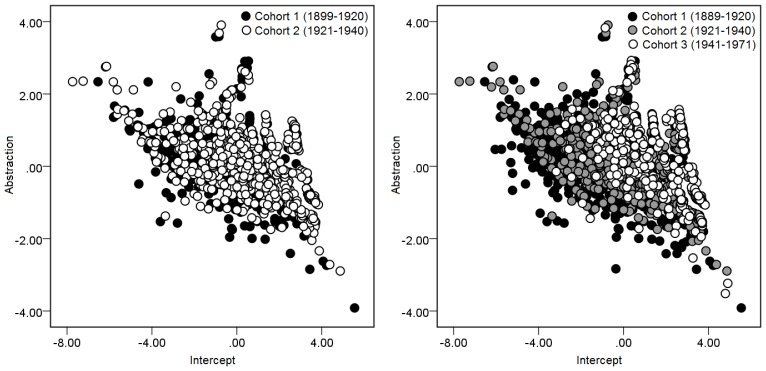
Abstraction as a function of intercept and cohort. Members of more recent cohorts tend to score higher on the abstraction variable than members of earlier cohorts who achieve the same intercept. The left panel is confined to the age-matched comparison between cohorts 1 and 2, whereas the right panel includes participants who were too old or young at the time of testing to be included in an age-matched comparison. Note that the results are in no way embellished by the parameter estimation process because cohort was never entered into the model.

The left panel of Figure 1 provides a graphical illustration of this difference with abstraction on the *x*-axis and the intercept on the *y*-axis. It can be seen that the white dots representing members of cohort 2 are positioned somewhat higher than the black dots representing members of cohort 1 for any given level of *x.* This result confirms that the Letter Series and Word Series tests violate measurement invariance between cohorts 1 and 2 with respect to the cognitive theory presented in this paper.

The aim of this study is not merely to test Fox and Mitchum’s [7] proposal with a comparison that does not confound cohort with age, but more generally, to determine how well it predicts differences between cohorts, irrespective of age as a confound. We present a similar scatterplot containing ability estimates of all participants, including those who were not included in the focal comparison of age-matched cohorts, in the right panel of Figure 1. This includes individuals who were too old to be included in cohort 1 (*n* = 483) or too young to be included in cohort 2 (*n* = 79) for the age-matched comparison, and cohort 3 participants who were born in the years 1941 through 1971.

The scatterplot of all three cohorts is especially striking in its revelation of changes across cohorts in the relative contributions of the two variables. It bears emphasis that because cohort itself was never specified in the model, the clear stratification by cohort observed in the scatterplot is a raw reflection of differences in item-response behavior across cohorts as predicted in this paper that is embellished in no way by the parameter estimation process (which necessarily entails taking for granted that a model is true, leading to “cleaner” looking findings). It would be clear even without conducting a statistical analysis that abstraction scores for any given level of the intercept increase across the three cohorts as evidenced by the clear stratification of individuals into cohorts, *t*(1) = 20.10, *p*<.001.

## Discussion

The aim of this study was to further test a cognitive account of cohort differences [7] that was originally presented and tested in the context of matrix reasoning against records of item-specific pass rates and the item responses of a contemporary sample of older and younger adults. The Long Beach Longitudinal Dataset made it possible to test the same proposal against a dataset that contains item responses to two different inductive reasoning tests and enables the effect of age group to be held constant. We predicted that that members of later cohorts would score higher on a latent variable defined by the level of abstraction at which objects must be mapped compared to members of earlier cohorts who achieve the same overall level of performance. This predicted violation of measurement invariance was confirmed, suggesting that Fox and Mitchum’s [7] proposal generalizes beyond the Raven’s Matrices to other inductive reasoning tests, and that Fox and Mitchum [7] were correct to attribute their findings to cohort rather than age. These results have implications for the Flynn effect and highlight the importance of establishing psychological criteria for equating members of distinct groups who achieve the same scores.

To fully appreciate the theoretical and methodological significance of our finding is to understand that it challenges a foundational assumption behind the use of tests in general as this assumption applies to using Letter and Word Series tests to compare members of different cohorts. Virtually all psychometric models, from the simple general linear model to complex latent variable models *take for granted* that a score on a test can be accurately interpreted as placement along one and only one dimension. To anyone who interprets these models as reflections of something psychological rather than as mere mathematical abstractions, the implication is that a given score has one psychological interpretation for everyone, irrespective of group membership. In contrast to this assumption, our analysis reveals that the psychological interpretation of a given score on the Letter and Word Series tests depends on the cohort of the individual who achieved that score.

It is necessary to consider two inherent limitations of the materials that constrained the size of the effect, namely, the briefness of the interlude between cohorts 1 and 2, and the less-than-optimal compatibility between the theory and the item features of the already-existing tests. Twenty years is an exceptionally short period of time to observe a change in the way individuals vary from one another within *the same* population, a subtle phenomenon that is considerably more difficult to detect than the phenomenon of rising scores itself. Indeed, 20 years is less than the time separating the births of the oldest and youngest siblings in many families. Another crucial constraint on the size of predicted effects is the imperfect design of the tests with respect to the conceptualization of item features in Fox and Mitchum’s [7] proposal. Although we were able to isolate three levels of abstraction, items could vary much more along this dimension in principle. Given these constraints, it could not have been taken for granted that the predicted effect would have been observed even if the veracity of Fox and Mitchum’s [7] proposal were already a given. Indeed, that the hypothesis was confirmed despite these constraints renders the proposal especially compelling.

Some may have noticed that although members of later cohorts obtained higher abstraction scores overall at a given level of the intercept, the highest levels of abstraction at any given level of the intercept are represented by both groups. This observation may seem relevant to the question of whether the Flynn effect is primarily a reflection of improvement at the lower end of the ability distribution as some have suggested [26]. Once again, we point to inherent limitations of defining variables within already existing tests. One potential limitation in this case is the relatively low level of abstraction needed to solve even the most difficult items, which could artificially constrain scores for some participants. Although our results clearly confirm our general prediction about violations of measurement invariance between cohorts and raise interesting questions for future research (which we discuss below), we recommend caution in drawing more specific inferences or generalizations.

### Implications for the Flynn Effect

There is still much to learn about the Flynn effect, but one major revelation of the last decade is that tests are often not measurement invariant over time [4], [5]. The present paper provides yet another example of a dataset in which persons vary differently from one another in two age-matched cohorts, but in addition to this, helps to paint a clearer picture of the cognition of rising scores over the 20th century. It is already possible to predict which tests will show large Flynn effects based on content [27], but the present results suggest that it is now possible to predict even which items within relatively homogeneous tests show the largest effects in light of Fox and Mitchum’s [7] proposal. The same proposal enables construction of new tests that may make it possible to distinguish abilities that vary between cohorts from others that vary primarily within cohorts. Although there is still a great deal to be learned about the social, cultural, and educational causes of rising scores, researchers have made significant strides toward understanding the trend as a cognitive phenomenon, and the present paper helps to further this understanding.

### Implications for Cross-Sectional Comparisons in Cognitive Aging

Because the Long Beach Longitudinal data were collected in the context of cognitive aging research [8], the implications of our findings for cognitive aging merit special consideration. It is common to draw inferences about the effect of aging on cognitive ability from cross-sectional comparisons of older and younger adults, that is, members of different cohorts, without testing for violations of measurement invariance between these groups. This is problematic in light of the violations of measurement invariance between cohorts observed in this paper and others discussed above. One reason for this neglect may be the existence of confirmatory factor-analytic studies confirming to a greater or lesser degree the similarity of variation in younger and older adult populations [28], [29]. However, as Fox and Mitchum [7] point out, a purely statistical approach to establishing measurement invariance is less able to uncover differences like the one reported in this paper. A useful analogy is to consider trying to find a lost item, such as a key, in a large field at night using only a fixed amount of light. One could distribute the light over the whole field, which would offer little illumination in any one place, or concentrate the light in the area where they believe the lost item is most likely to be found. If light is analogous to statistical power, then the former approach is analogous to an exploratory test of measurement invariance, whereas the latter is analogous to the more theoretical and confirmatory approach taken in this paper. The strongest inferences about the psychological sameness of members of two groups who achieve the same score require actually modeling the psychological processes involved in selecting responses.

## Conclusion

In this paper we utilized a process-oriented psychometric methodology to test a cognitive theory of cohort differences [7] against a dataset that enabled the effect of cohort to be isolated. The Letter and Word Series tests allowed the proposal to be tested against two new tests. Even though the two age-matched cohorts were separated by only 20 years, predicted violations of measurement invariance were observed as members of the later cohort were found to map objects at higher levels of abstraction than members of earlier cohort who possess the same overall ability. Our results confirm predicted differences between cohorts in patterns of between-subjects variation, as illustrated by the stratification of cohorts in Figure 1, and verify that Fox and Mitchum’s [7] proposal generalizes beyond the Raven’s Matrices to several other inductive reasoning tests.

One major advantage of this process-oriented framework is its provision of independent, psychological (as opposed to merely psychometric) criteria for comparing variation in two cohorts, which obviates the statistical essentialism of equating psychological properties with variation itself. That individuals vary from one another somewhat differently between cohorts than within cohorts implies that the distributions of various test-relevant psychological processes and competencies are different between cohorts than within cohorts. Between-subjects variables defined by statistical procedures (e.g., psychometric-*g,* fluid intelligence, etc.) rather than actual psychological criteria are of little relevance to understanding a difference between groups simply because their psychological interpretation depends on the groups in which they are observed [30]. In contrast, the processes utilized by a person to select an item response are relevant because their psychological interpretation does not depend on group membership. Thus, our findings are not only an empirical contribution but an illustration of how questions of psychological equivalence can be informed by an approach that fully incorporates both theory and method.

Indeed, we believe that as the apparent paradox of rising scores continues to unravel in the coming years, the most profound upshot will be not merely a fuller understanding of how and why cognition changed during the 20^th^-century, but greater awareness of why an empirical science of individual differences must rest upon a sound conceptual and theoretical foundation.
